# A Novel Prognostic Predictor of Immune Microenvironment and Therapeutic Response in Kidney Renal Clear Cell Carcinoma based on Necroptosis-related Gene Signature

**DOI:** 10.7150/ijms.69060

**Published:** 2022-01-24

**Authors:** Wenwei Chen, Wenfeng Lin, Liang Wu, Abai Xu, Chunxiao Liu, Peng Huang

**Affiliations:** 1 Department of Urology, Zhujiang Hospital, Southern Medical University, Guangzhou, China.; 2 Department of Urology & Department of Kidney Transplantation, The First Affiliated Hospital, Wenzhou Medical University, Wenzhou, China.; 3 Department of Urology, Okayama University Graduate School of Medicine, Dentistry and Pharmaceutical Sciences, Okayama, Japan.; 4 Department of Pathology, The First Affiliated Hospital, Wenzhou Medical University, Wenzhou, China.

**Keywords:** prognosis, immune microenvironment, therapeutic response, kidney renal clear cell carcinoma, necroptosis, gene signature

## Abstract

**Background:** Necroptosis, a cell death of caspase-independence, plays a pivotal role in cancer biological regulation. Although necroptosis is closely associated with oncogenesis, cancer metastasis, and immunity, there remains a lack of studies determining the role of necroptosis-related genes (NRGs) in the highly immunogenic cancer type, kidney renal clear cell carcinoma (KIRC).

**Methods:** The information of clinicopathology and transcriptome was extracted from TCGA database. Following the division into the train and test cohorts, a three-NRGs (TLR3, FASLG, ZBP1) risk model was identified in train cohort by LASSO regression. The overall survival (OS) comparison was conducted between different risk groups through Kaplan-Meier analysis, which was further validated in test cohort. The Cox proportional hazards regression model was introduced to assess its impact of clinicopathological factors and risk score on survival. ESTIMATE and CIBERSORT algorithms were introduced to evaluate immune microenvironment, while enrichment analysis was conducted to explore the biological significance. Correlation analysis was applied for the correlation assessment between checkpoint gene expression and risk score, between gene expression and therapeutic response. Gene expressions from TCGA were verified by GEO datasets and immunohistochemistry (IHC) analysis.

**Results:** This NRGs-related signature predicted poorer OS in high-risk group, which was also verified in test cohort. Risk score could also independently predict survival outcome of KIRC. Significant changes were also found in immune microenvironment and checkpoint gene expressions between different risk groups, with immune functional enrichment in high-risk group. Interestingly, therapeutic response was correlated with the expressions of NRGs. The expressions of NRGs from TCGA were consistent with those from GEO datasets and IHC analysis.

**Conclusion:** The NRGs-related signature functions as a novel prognostic predictor of immune microenvironment and therapeutic response in KIRC.

## Introduction

Kidney cancer incidence is rising globally, particularly in the younger population 1, 2. In 2020, there are more than 431,000 new cases and 179,000 deaths from kidney cancer in the world 3. Kidney renal clear cell carcinoma (KIRC), the most common histological subtype, is characterized by extensive tumor heterogeneity, distinct clinical courses, and potential specific treatment vulnerabilities 4. Genetic alterations occur frequently in KIRC, such as somatic mutations of VHL, PBRM1, SETD2, BAP1, KDM5C, and PI3K-AKT-mTOR pathway genes 4, 5. Although nephrectomy partially or radically showed good efficacy in treating localized KIRC, over 30% patients present with advanced-stage disease, and 25% of all patients ultimately experience disease relapse 6. In recent years, the treatment scenario of advanced KIRC has evolved dramatically, with the emergence of targeted agents and immune checkpoint inhibitors (ICI). Indeed, real-life clinical practice faces the huge challenge of optimizing individualized therapeutic strategies. Biomarkers and prediction models can be applied for improving risk stratification and case selection for targeted therapy, immunotherapy, and combined therapeutics 4. To date, no reliable predictive biomarkers have been identified for mirroring immune microenvironment and therapeutic response in KIRC.

Necroptosis is a caspase-independent necrotic cell death by genetical regulation, the main mediators of which include receptor interacting protein kinase 1 (RIPK1) and RIPK3, and mixed lineage kinase domain-like (MLKL) 7. Accumulating evidence indicates that necroptosis plays a critical role in regulating oncogenesis and cancer progression, but it seems to be a double-edged sword 8. For instance, the key mediator of necroptotic pathway, RIPK3 is downregulated in several cancer types, which associates with the increased tumor aggressiveness and chemoresistance 9-11. This evidence suggests that necroptosis plays a positive role in anti-cancer progression. Conversely, the key necroptotic executioner, MLKL is upregulated in some cancers, correlating with highly invasive tumor behavior and immunosuppressive microenvironment 12, 13. In addition, experimental studies indicate that cancer cells can induce the necroptosis of microvascular endothelial cells, thus promoting cancer cell extravasation and metastasis 14, 15. Therefore, the function of necroptosis in cancer development is complex and context-dependent.

Interestingly, the spontaneous and mild necroptosis of tumor cells triggers pro-tumor immunity through releasing immunosuppressive molecules to modulate the tumor microenvironment including myeloid-derived suppressor cells (MDSC) and M2-like macrophages 16. In contrast, the massive and acute necroptosis of tumor cells induced by chemotherapy or irradiation is often more immunogenetic, increasing the anti-tumor immunity through the activation of IFN-γ-expressing T cells 16. Necroptosis has emerged as a promising cancer therapeutic target in combination with cancer immunotherapy. In the murine model, necroptosis induction of tumor cells *in situ*, can improve anti-tumor immunity when synergized with immune checkpoint blockade 17, 18. Numerous key molecules in necroptotic pathways have been identified to be critical factors for cancer prognosis. The decreased MLKL expression is correlated with a reduced overall survival (OS) in ovarian carcinoma, gastric cancer, and colon cancer 19-21. The upregulated RIPK1 expression associates with a poor prognosis in glioblastoma and breast cancer 22, 23.

**Figure 1:**
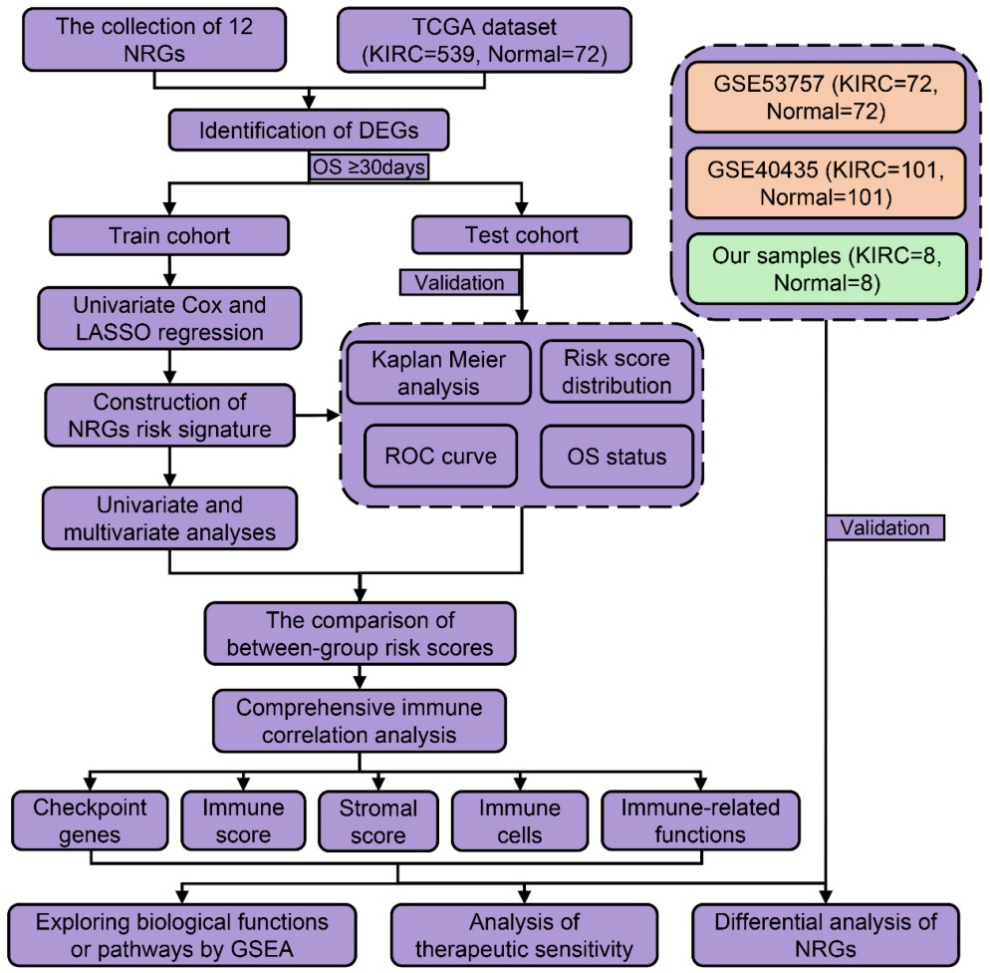
The flow process diagram of identifying NRGs risk model.

However, it remained unclear whether necroptosis-related genes (NRGs) were associated with KIRC prognosis. This study was designed to construct a NRGs risk model in KIRC and determine its relationship with tumor immunity and therapeutic response. As presented in the flow process diagram **(Figure 1)**, we developed a three-NRGs (TLR3, FASLG, ZBP1) signature which could predict OS, immune microenvironment, and therapeutic response in KIRC, followed by the verification of TCGA test cohort, GEO datasets, and clinical histological staining.

## Materials and methods

### Data acquisition

The collection of 12 necroptosis-related genes (NRGs) was conducted from the platform “http://www.gsea-msigdb.org/gsea/msigdb/search.jsp”, including CASP8, CFLAR, FADD, FAS, FASLG, MLKL, RIPK1, RIPK3, TICAM1, TLR3, TNF, and ZBP1. TCGA database provided the information of clinicopathology and transcriptome with KIRC samples (T) = 539 and normal samples (N) = 72. GEO database provided two datasets including GSE40435 (N = 101, T = 101), and GSE53757 (N = 72, T = 72).

### Construction of a prognostic NRGs signature and its validation

The expression data of 12 NRGs were firstly extracted from TCGA database, followed by the screening of differentially expressed genes (DEGs) between KIRC and normal tissues using R package “limma” with the filter conditions (fdrFilter = 0.05, logFCfilter = 1). After integrating the expression data of DEGs and survival data (more than 30 days), R package “caret” was applied to divide them into two cohorts (train cohort and test cohort). In train cohort, R package “glmnet” and the least absolute shrinkage and selection operator (LASSO) regression were applied for the NRGs risk signature identification. Following the risk score calculation using the formula: risk score = ∑ (gene expression × corresponding regression coefficient) 24, the subjects were classified into high-risk and low-risk groups based on the median score. R packages “survival” and “survminer” were introduced to evaluate overall survival (OS) based on Kaplan Meier (K-M) method. R package “timeROC” was applied for the generation of receiver operating characteristic (ROC) curve, while the area under the ROC curves (AUCs) of risk score, grade, and stage were used to evaluate the accuracy for predicting OS. Principal component analysis (PCA) was introduced for the exploration of group distribution using R package “ggplot2”. Univariate and multivariate Cox proportional hazards regression models were introduced to evaluate the impact of risk score, and clinicopathological factors (age, gender, pathological grade, and clinical stage) on OS. The same analyses were conducted to validate the risk signature power in the test cohort. GSE40435 and GSE53757 datasets were further introduced to verify the expressions of FASLG, TLR3, and ZBP1.

### Immune microenvironment assessment

The immune infiltration (immune score and stromal score) was assessed by the ESTIMATE algorithm 25, followed by analyzing the correlation of immune infiltration with risk score.

### Immune enrichment analysis

Single sample gene set enrichment analysis (ssGSEA) was applied for the score calculation of immune cells and immune-related functions between different risk groups using R package “GSVA”. As the annotated reference, “c5.all.v7.4.symbols.gmt” was introduced to GSEA software (v 4.1.0) for the exploration of potential immunomodulatory functions.

### Drug response analysis

From the CellMiner platform, we obtained the NCI-60 data, containing 60 types of cancer cell lines and the efficacy of FDA-approved drugs. The correlation of NRGs expressions with therapeutic response was assessed using Pearson correlation test.

### Immunohistochemistry (IHC) staining

Eight pairs of paraffin-embedded KIRC and adjacent samples were collected in the First Affiliated Hospital of Wenzhou Medical University. Slices were baked at 65 °C for 2 h, followed by dewaxing and antigen retrieval. After 10-min inactivation of endogenous enzymes by 3% hydrogen peroxide at room temperature, the slices underwent PBS rinsing 3 times for 3 min each. Following the blocking step by bovine serum albumin, the primary antibodies against FASLG, TLR3, and ZBP1 were applied to incubate the slices overnight at 4 °C. Then washed them with PBS 3 times for 5 min each. After incubation with the secondary antibody at 37 °C for 30 min, the slices underwent DAB color rendering for 5-10 min, and hematoxylin redye for 3 min. The slices were finally observed under microscope, followed by integrated optical density (IOD) measurement by Image Pro Plus 6.0 image software. The relative expressions of FASLG, TLR3, and ZBP1 were presented as average optical density (IOD/positive staining area).

### Statistical analysis

R software (v 4.1.0), IBM SPSS software (v 22), and GraphPad Prism (v 8.3) were applied for all statistical analyses and diagram drawing. Chi-Square test and K-M method were used to compare the characteristics in **Table 1** of train cohort, and test cohort. The survival comparison and independent OS predictors were analyzed using K-M method, univariate, and multivariate Cox proportional hazards regression model, respectively. Pearson or Spearman correlation test was applied to analyze the correlation concerning risk score. The two-way analysis of variance (ANOVA) in GraphPad Prism was conducted for the comparison of IHC quantitative results. P values less than 0.05 were identified as statistical differences.

## Results

### Identification of differentially expressed NRGs with prognostic value

**Figure 2:**
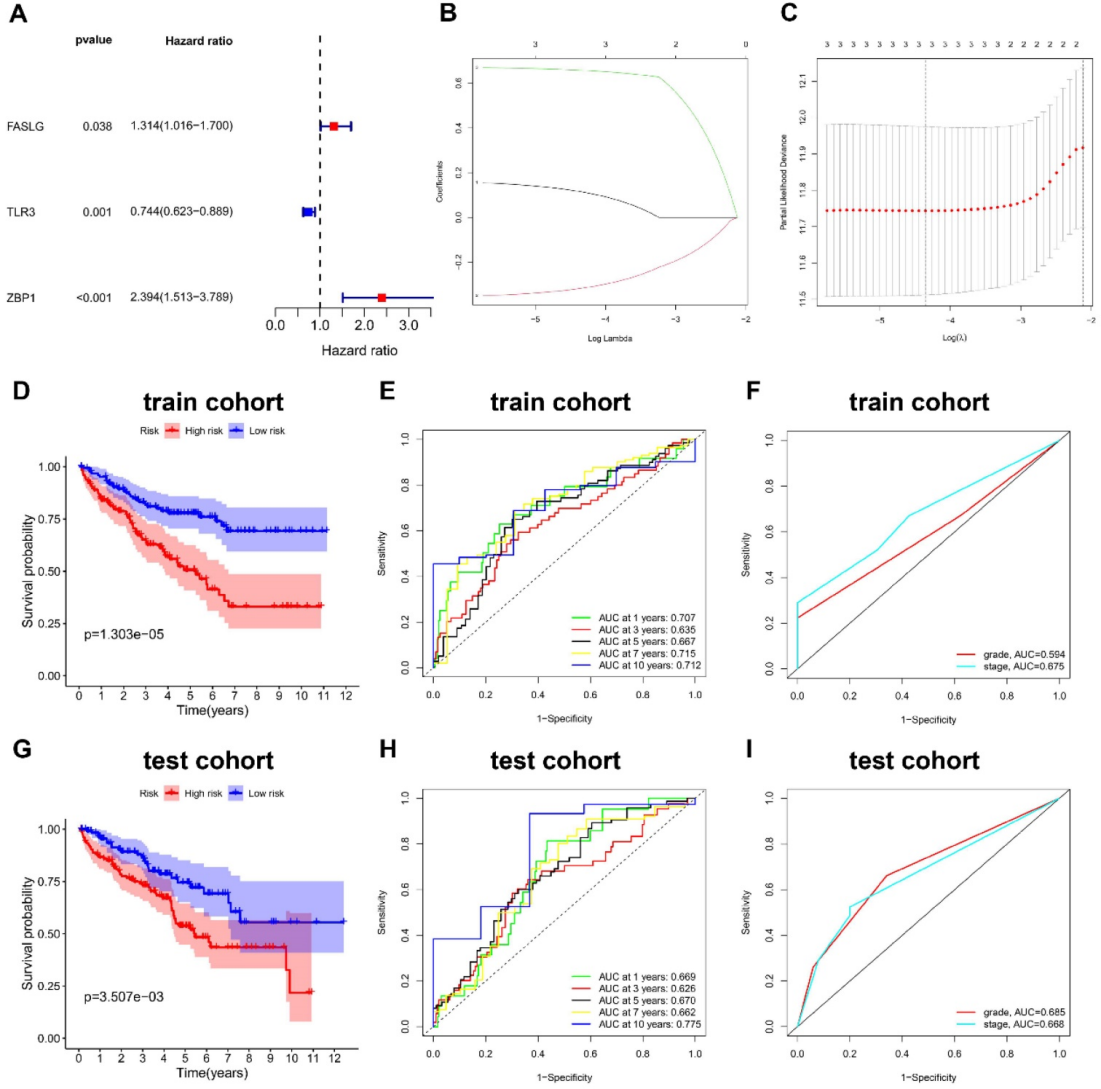
** The NRGs risk model construction in KIRC. (A)** The NRGs with prognostic values assessed by univariate Cox proportional hazards regression model in train cohort. **(B-C)** The selection of three-NRGs for risk model by LASSO analysis. K-M curves and time-dependent ROC curves for OS in **(D-F)** train cohort and **(G-I)** test cohort. Abbreviation: KIRC, kidney renal clear cell carcinoma; NRGs, necroptosis-related genes; LASSO, the least absolute shrinkage and selection operator; K-M, Kaplan-Meier; OS, overall survival.

We firstly divided 513 KIRC patients with survival time more than 30 days into train cohort (N = 257) and test cohort (N = 256). **Table 1** shows the characteristics of train cohort, test cohort, and entire cohort. From 12 NRGs mentioned above, we then screened 5 DEGs (FAS, FASLG, MLKL, TLR3, and ZBP1) between KIRC and normal tissues. Among these DEGs, we further identified three prognostic genes in train cohort, including FASLG (hazard ratio, HR = 1.314; 95% confidence interval, 95%CI = 1.016-1.700; P = 0.038), TLR3 (HR = 0.744; 95%CI = 0.623-0.889; P = 0.001), and ZBP1 (HR = 2.394; 95%CI = 1.513-3.789; P < 0.001) **(Figure 2A)**.

### Construction and validation of the NRGs risk signature

In train cohort, LASSO regression model analysis was conducted on the remaining prognostic NRGs (FASLG, TLR3, and ZBP1), which finally confirmed the least errors of three NRGs including FASLG, TLR3, and ZBP1 in the risk signature **(Figure 2B-2C)**. The formula was listed as follows: risk score = (0.115 × FASLG expression) + (-0.316 × TLR3 expression) + (0.658 × ZBP1 expression). K-M method was performed to determine the impact of risk signature on prognosis, finding that KIRC patients in low-risk group had a longer OS compared with those in high-risk group (P < 0.001) **(Figure 2D)**. The AUCs of risk score for predicting 1/3/5/7/10-year OS were 0.707, 0.635, 0.667, 0.715, and 0.712, respectively **(Figure 2E)**. The AUCs of grade and stage for predicting 10-year OS were 0.594 and 0.675, respectively **(Figure 2F)**. The same coefficients were applied for the risk score calculation in test cohort. Consistently, test cohort showed the similar result in survival outcome (P < 0.01) **(Figure 2G)**. The AUCs of risk score for predicting 1/3/5/7/10-year OS were 0.669, 0.626, 0.670, 0.662, and 0.775, respectively **(Figure 2H)**. The AUCs of grade and stage for predicting 10-year OS were 0.685 and 0.668, respectively **(Figure 2I)**. The risk score formula was also applied to predict OS between high- and low-risk groups in other subtypes of renal cell carcinoma (RCC), such as kidney renal papillary cell carcinoma (KIRP) and kidney chromophobe (KICH), which showed a poorer ability in predicting OS than KIRC subtype **(Figure S1)**.

**Figure 3:**
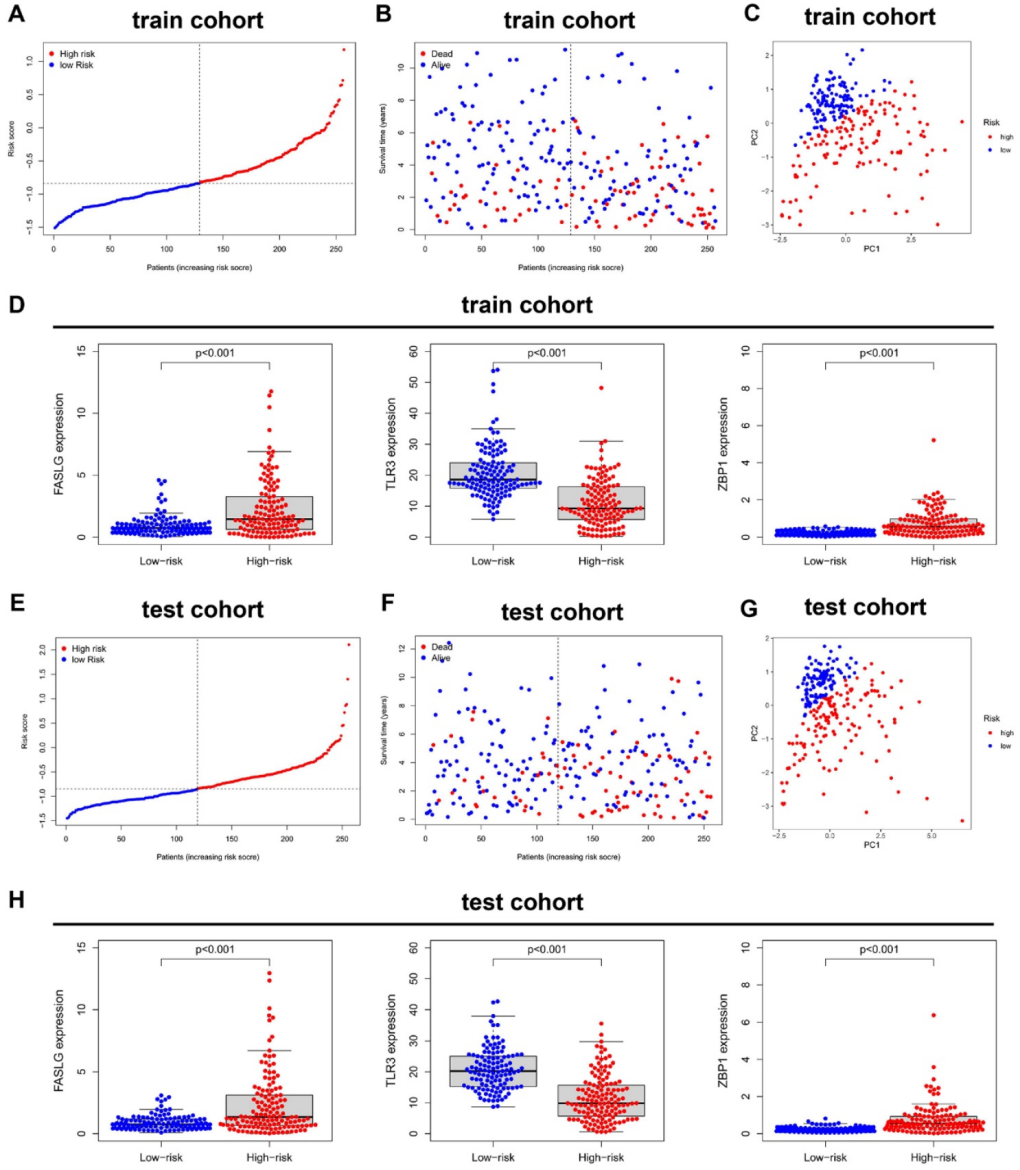
** Validation of this NRGs-related risk model. (A)** The risk score distribution and **(B)** OS status in train cohort. **(C)** PCA plot and **(D)** the comparison of FASLG, TLR3, and ZBP1 expressions between high- and low-risk groups in train cohort. **(E)** The risk score distribution and **(F)** OS status in test cohort. **(G)** PCA plot and** (H)** the comparison of FASLG, TLR3, and ZBP1 expressions between high- and low-risk groups in test cohort.

In train cohort, the distribution of ranked risk scores and individual survival status were shown in **Figure 3A, 3B**, indicating a longer survival time for KIRC patients in low-risk group. Moreover, the between-group distribution was discrete in PCA scatter plot **(Figure 3C)**. The comparisons in **Figure 3D** suggested the upregulated expressions of FASLG and ZBP1, but the downregulated expression of TLR3 in high-risk group. Consistently, the results including risk score distribution, individual survival status, PCA scatter plot, and gene expression comparisons were all successfully validated in test cohort **(Figure 3E-3H)**.

### The risk score in three-NRGs signature independently predicts OS in KIRC

**Figure 4:**
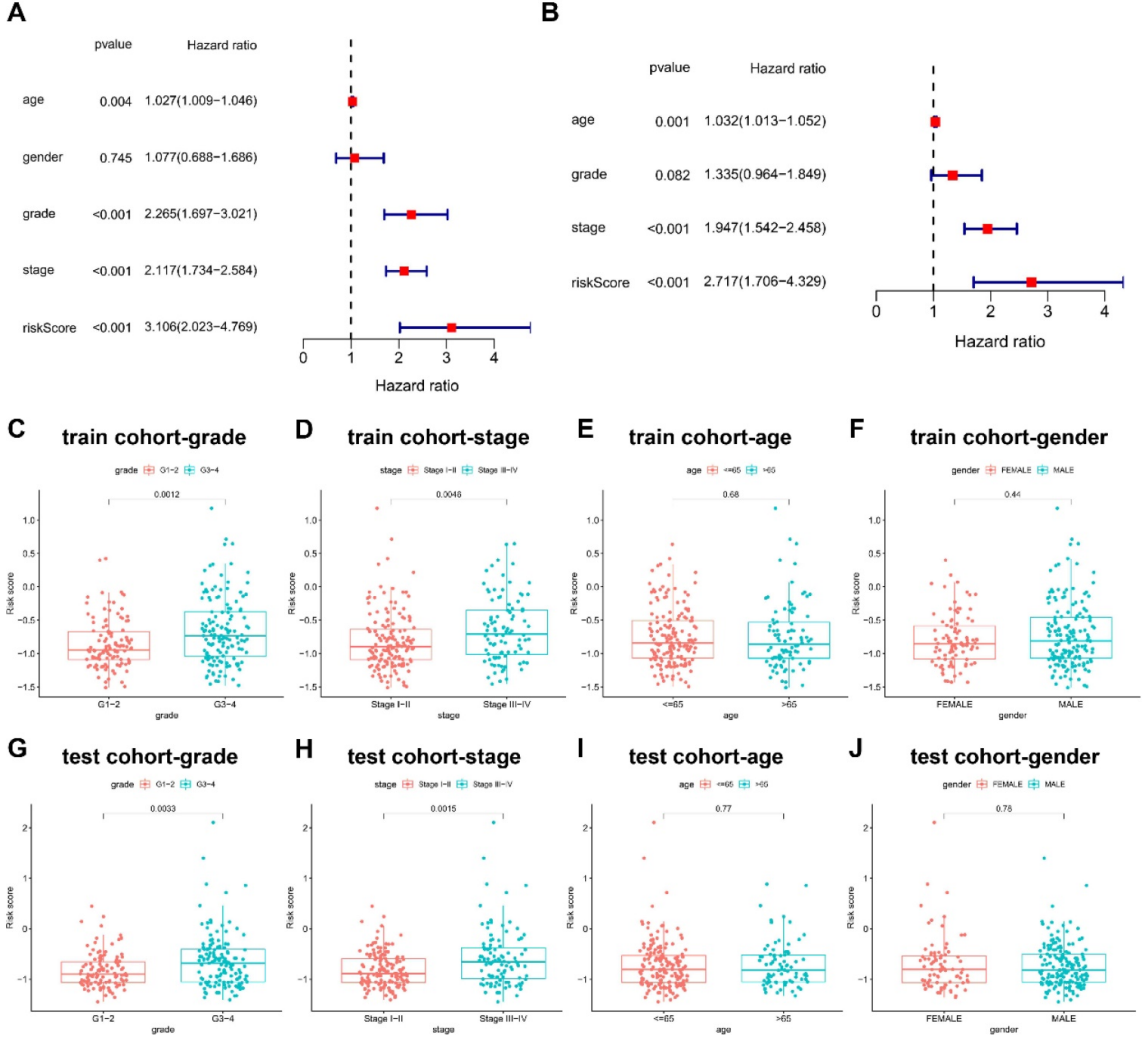
** Prognostic values of clinicopathological factors and risk score. (A-B)** Screening the independent predictors for OS in KIRC by univariate and multivariate Cox proportional hazards regression model. Since univariate regression analysis indicated no statistical difference in gender, it was not included in multivariate Cox proportional hazards regression model. The comparison of risk score between groups based on **(C)** pathological grade, **(D)** clinical stage, **(E)** age, and **(F)** gender in train cohort. The comparison of risk score between groups based on **(G)** pathological grade, **(H)** clinical stage, **(I)** age, and **(J)** gender in test cohort.

Cox proportional hazards regression model was introduced for the screening of the OS independent predictors. In univariate analysis, the factors with significant difference were listed as follows: risk score (HR = 3.106; 95% CI = 2.023-4.769; P < 0.001), clinical stage (HR = 2.117; 95%CI = 1.734-2.584; P < 0.001), and pathological grade (HR = 2.265; 95%CI = 1.697-3.021; P < 0.001) **(Figure 4A)**. Univariate regression analysis indicated no statistical difference in gender which was not included in multivariate Cox proportional hazards regression model. Therefore, multivariate Cox proportional hazards regression model was further applied to analyze these significant factors, finding that the independent predictors for OS included risk score (HR = 2.717; 95%CI = 1.706-4.329; P < 0.001), clinical stage (HR = 1.947; 95%CI = 1.542-2.458; P < 0.001) **(Figure 4B)**.

Differential risk score was further compared between groups based on clinicopathological factors. G3-4 and stage III-IV groups had higher risk scores than G1-2 (P < 0.01) and stage I-II (P < 0.01), but no statistical difference was found in risk scores between groups of age and gender in train cohort **(Figure 4C-4F)**, which was successfully validated in test cohort **(Figure 4G-4J)**.

### The NRGs signature closely associates with tumor immunity

**Figure 5:**
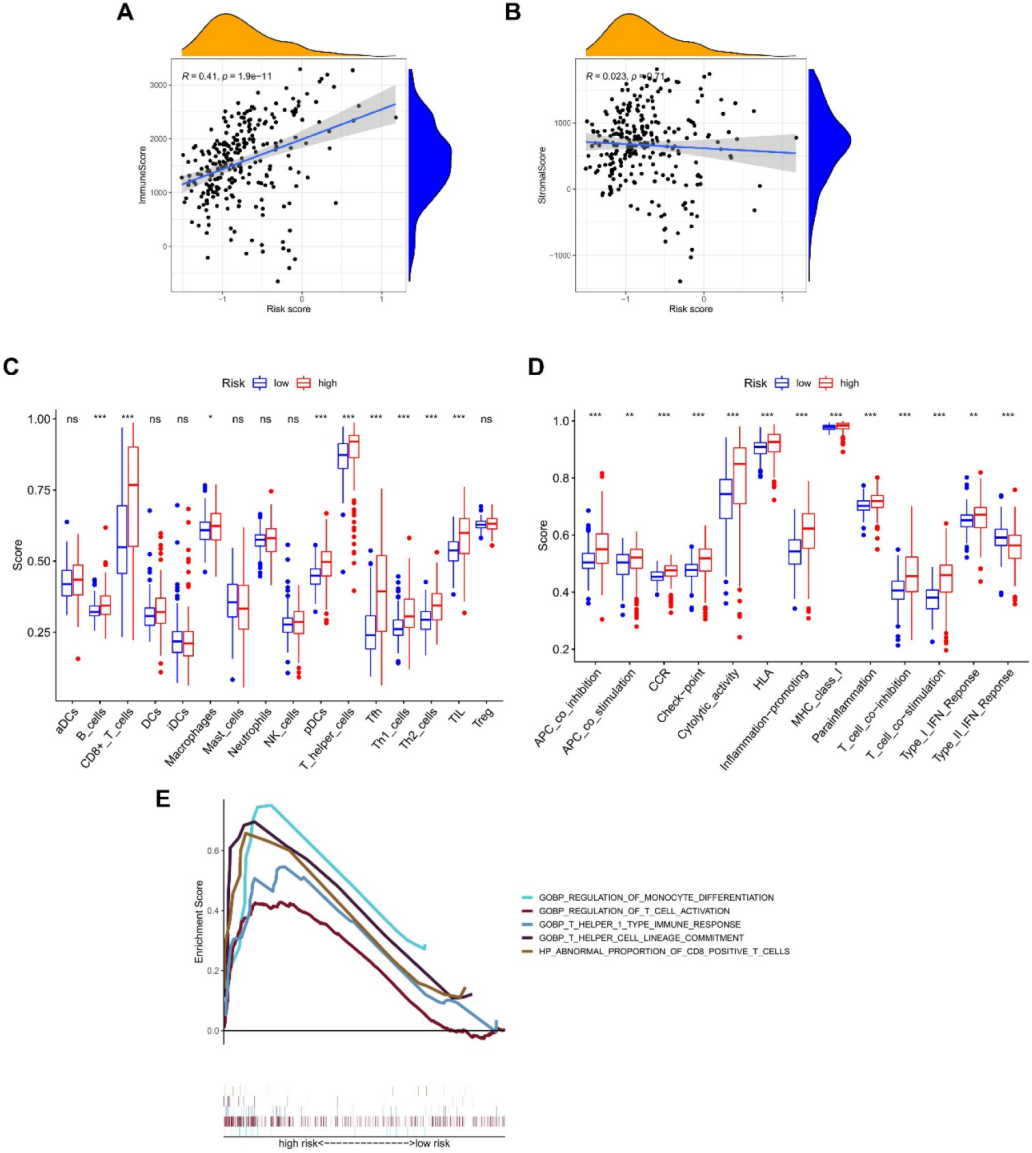
** Correlation of NRGs-related signature with immune microenvironment.** The association between risk score and **(A)** immune score, or **(B)** stromal score. Boxplots comparing the scores of **(C)** immune cells and **(D)** immune-related functions between different risk groups. **(E)** Immune functions enriched in high-risk group.

**Figure 6:**
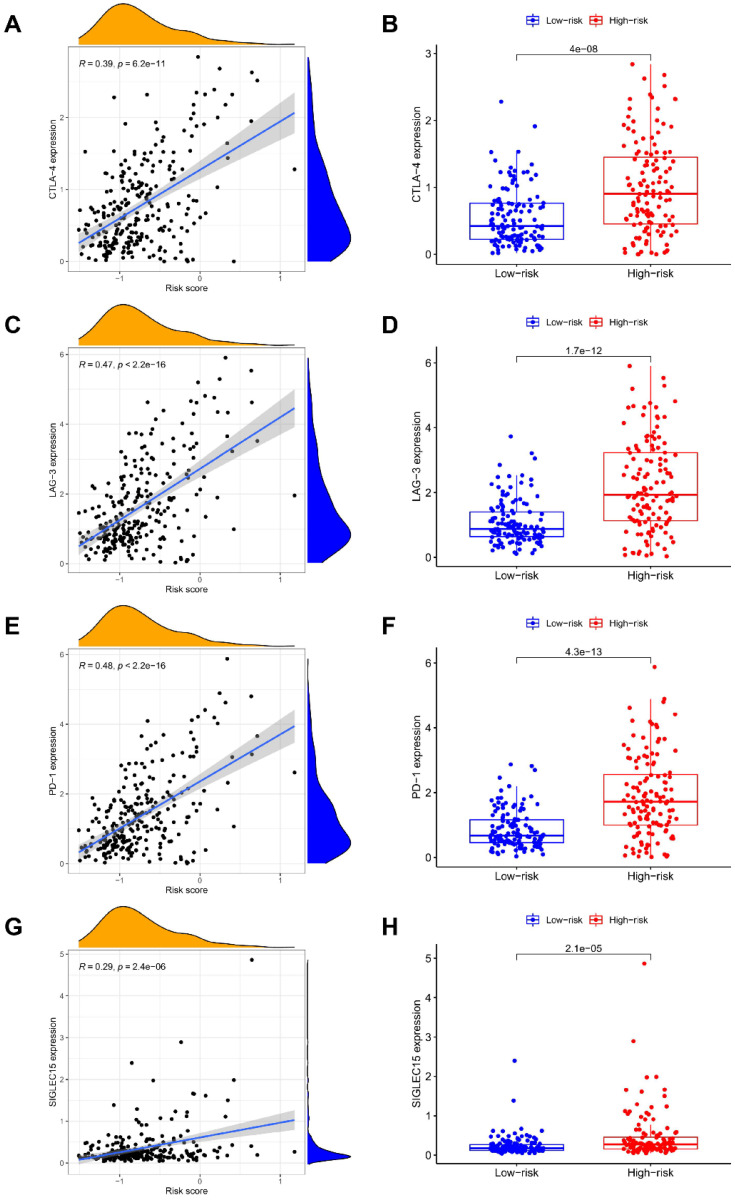
** Correlation of NRGs-related signature with checkpoint gene expressions.** The association of risk score with the expression levels of **(A-B)** CTLA-4, **(C-D)** LAG-3, **(E-F)** PD-1, and **(G-H)** SIGLEC15.

The ESTIMATE algorithm was introduced to assess immune infiltration, showing a positive correlation of risk score with immune score **(**P < 0.001; **Figure 5A)**, but no statistical difference was found between risk score and stromal score **(Figure 5B)**. The score comparisons were performed in 16 immune cell types and 13 kinds of immune-related functions between different risk groups. Among these immune cells, 9 types including B cells, CD8 positive T cells, Macrophages, pDCs, T helper cells, Tfh, Th1 cells, Th2 cells, and TIL, had higher scores in high-risk group than in low-risk group **(Figure 5C)**. Among these immune-related functions, 12 kinds including Parainflammation, MHC class I, inflammation promoting, APC co-inhibition or co-stimulation, check point, Cytolytic activity, CCR, T cells co-inhibition or co-stimulation, HLA, and Type I IFN Response, had higher scores in high-risk group than in low-risk group, except Type II IFN Response **(Figure 5D)**. We further enriched the immunomodulatory functions, including regulation of monocyte differentiation, regulation of T cell activation, T helper 1 type immune response, T helper cell lineage commitment, and abnormal proportion of CD8 positive T cells **(Figure 5E)**. In addition, the expressions of immune checkpoint genes (CTLA-4, LAG-3, PD-1, and SIGLEC15) were positively correlated with risk scores, and were significantly higher in high-risk group **(Figure 6A-6H)**.

### The expressions of NRGs associates with therapeutic response

**Figure 7:**
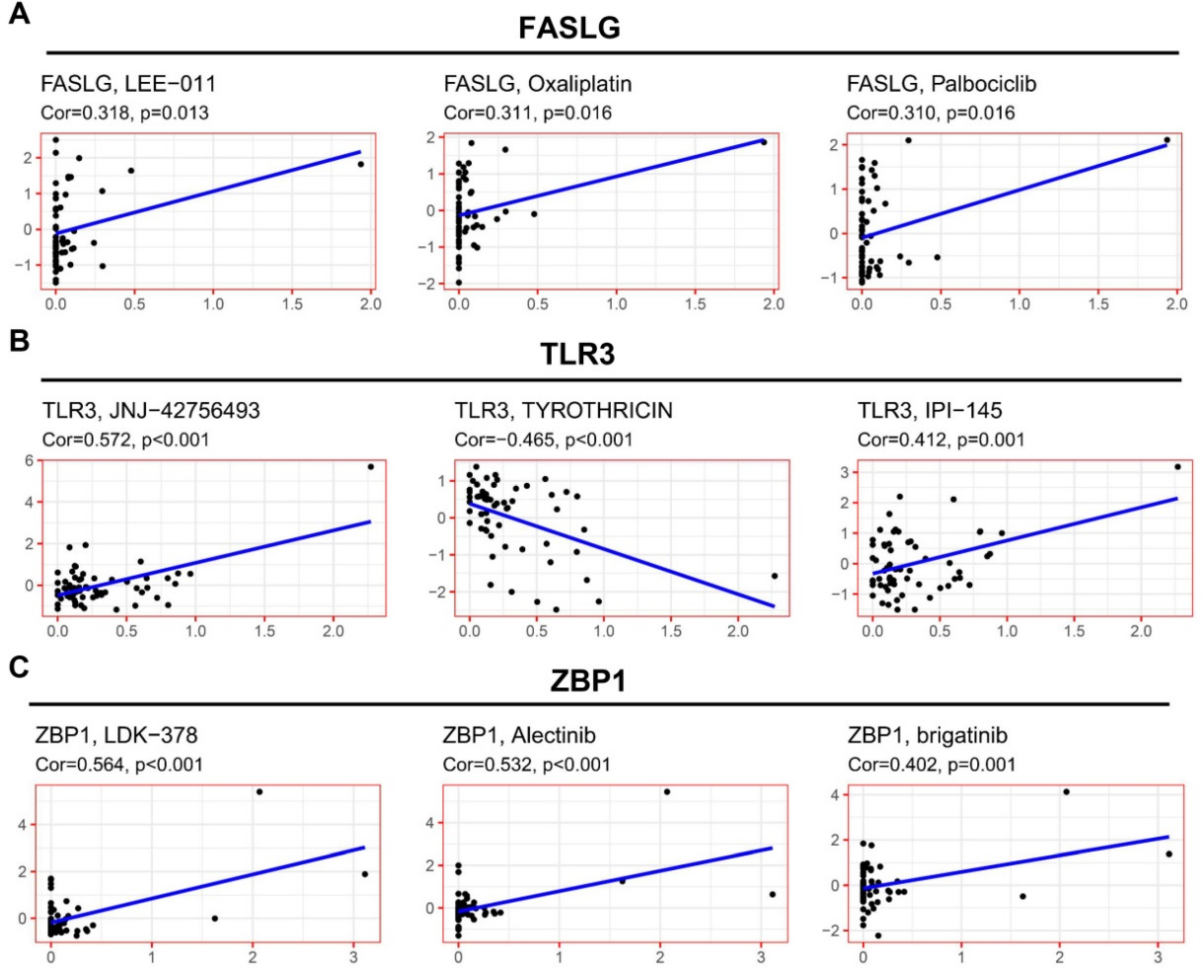
** Correlation of NRGs expressions with therapeutic response.** The association between drug sensitivity and the expressions of **(A)** FASLG, **(B)** TLR3, and **(C)** ZBP1.

To explore the potential clinical significance of NRGs signature, we integrated the data concerning cancer cell expressions and the efficacy of FDA-approved drugs. Cancer cells with higher FASLG expression were more sensitive to LEE-011, oxaliplatin, and palbociclib **(Figure 7A)**. Cancer cells with higher TLR3 expression were more sensitive to JNJ-42756493 and IPI-145 **(Figure 7B)**, but they were correlated with increased drug resistance to tyrothricin. Cancer cells with higher ZBP1 expressions were more sensitive to the following drugs including LDK-378, alectinib, and brigatinib **(Figure 7C)**.

### Differential expressions of NRGs between KIRC and normal samples

**Figure 8:**
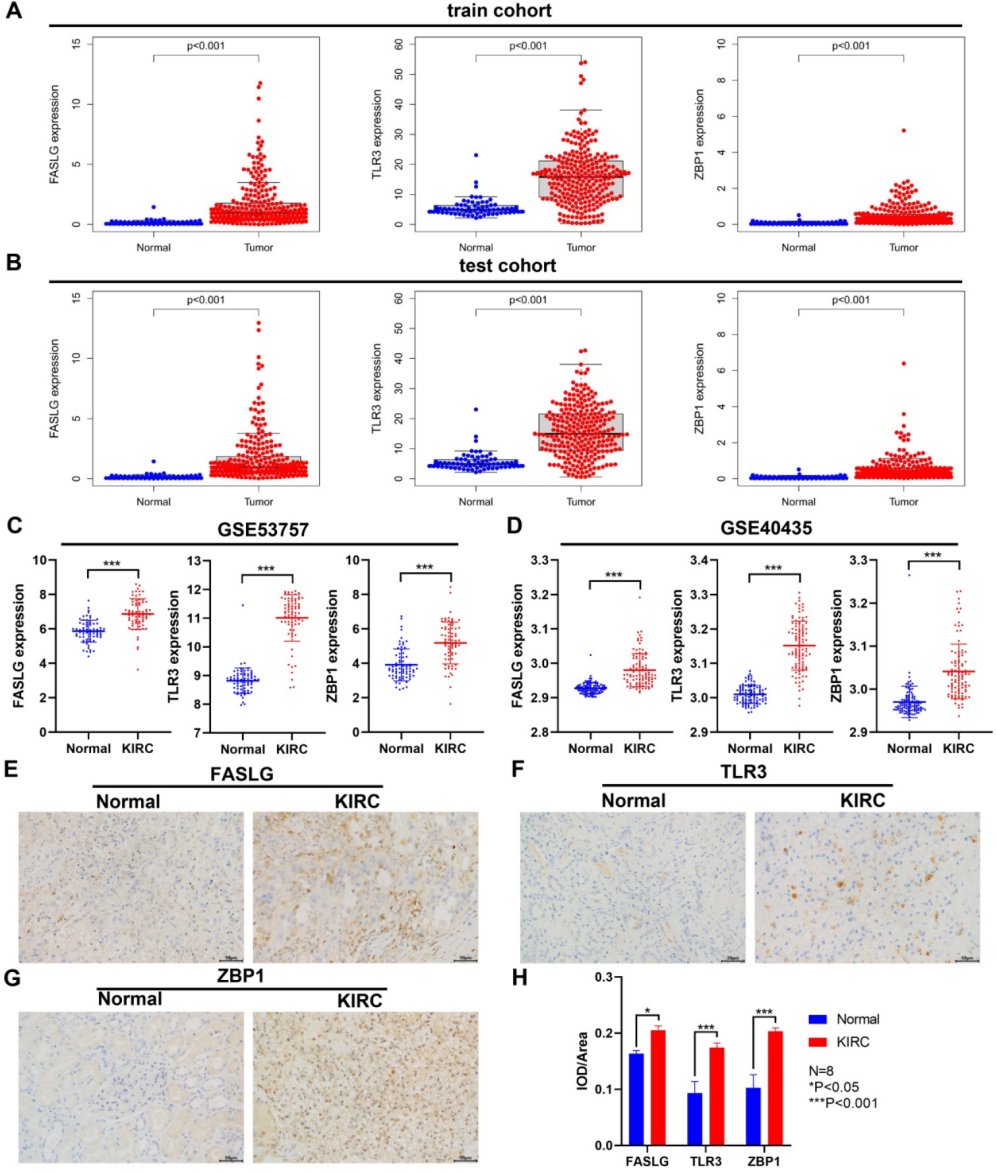
** Differential expressions of NRGs by GEO and IHC staining validation.** The comparison of FASLG, TLR3, and ZBP1 expressions between KIRC and normal samples in train cohort **(A)** and test cohort **(B)**. Differential expressions of FASLG, TLR3, and ZBP1 validated by GSE53757 **(C)** and GSE40435 **(D)** datasets. **(E-G)** IHC staining was applied to validate the differential expressions of FASLG, TLR3, and ZBP1 using KIRC samples from our clinical center. Scale bar = 50 µm. **(H)** Image Pro Plus 6.0 image software was applied to assess the relative expressions of FASLG, TLR3, and ZBP1 which were presented as average optical density. Data are expressed as mean ± SEM.

Through TCGA database, we found the significant upregulation of FASLG, TLR3, and ZBP1 expressions both in train cohort and test cohort **(**P < 0.001; **Figure 8A-8B)**. Consistently, in GSE53757 and GSE40435 datasets, the expressions of the three genes were significantly higher in KIRC tissues than in normal ones (P < 0.001; **Figure 8C-8D)**. At the histological level, we further used IHC staining to validate the differential expressions of FASLG, TLR3, and ZBP1 in KIRC samples **(Figure 8E-8H)**.

### The construction of a NRGs nomogram for predicting survival

**Figure 9:**
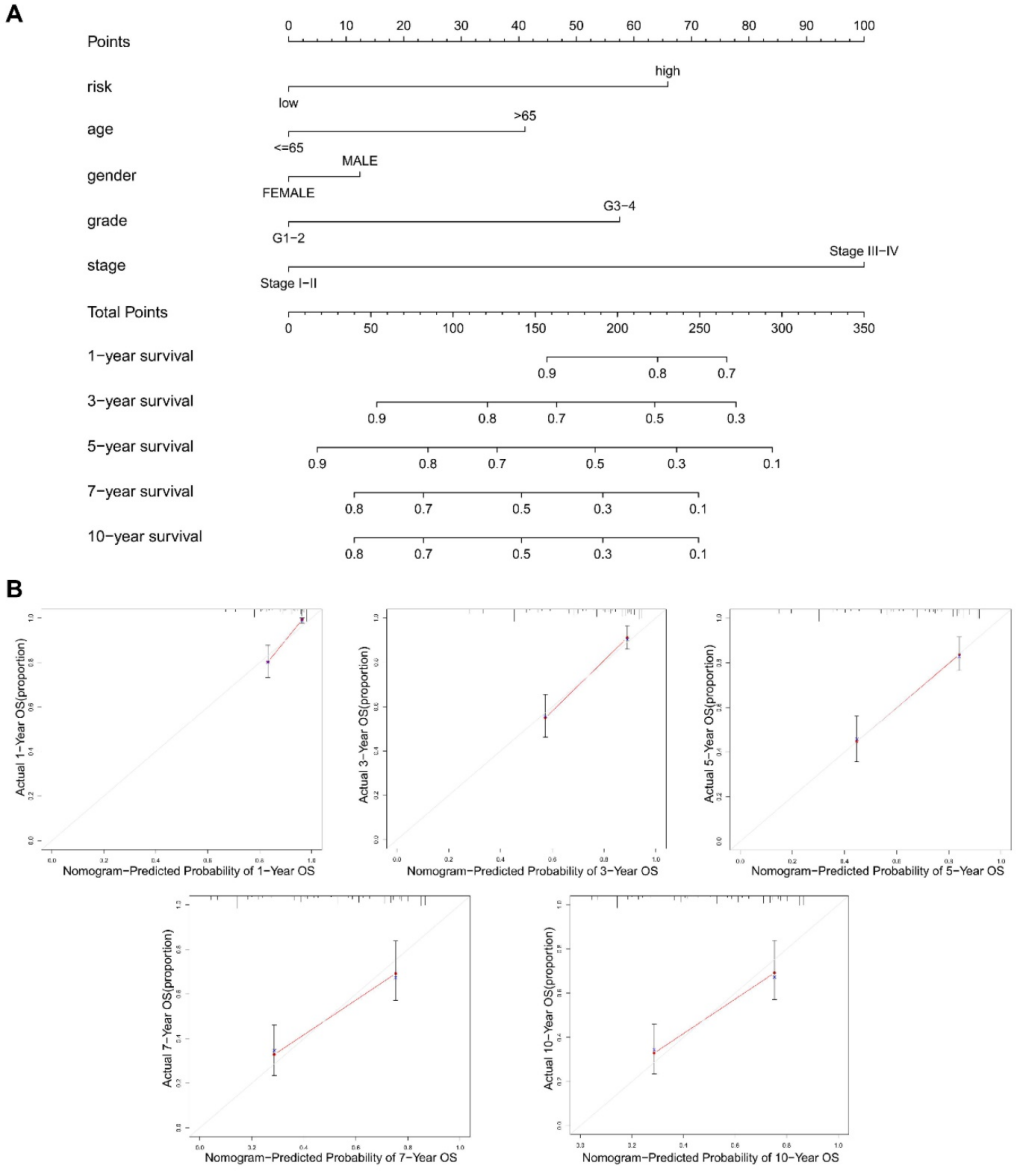
** The construction of a nomogram for predicting survival. (A)** A nomogram including risk score and clinicopathological features was constructed to predict 1/3/5/7/10-year OS. **(B)** The calibration plots for predicting 1/3/5/7/10-year OS based on the NRGs nomogram.

To promote the clinical value of this novel risk model, a nomogram including risk score and clinicopathological features was constructed to predict 1/3/5/7/10-year OS **(Figure 9A)**. The calibration plots for predicting 1/3/5/7/10-year OS based on the NRGs nomogram exhibited a favorable agreement of actual probability with the predicted probability **(Figure 9B)**. To highlight the role of risk score in this nomogram, we constructed a second nomogram including only clinicopathological features, showing the inferior prediction effect especially in predicting 7- and 10-year OS **(Figure S2)**.

## Discussion

Clear cell renal cell carcinoma (ccRCC), also known as KIRC, is featured by angiogenic, inflammatory, and highly immunogenic microenvironment, showing different sensitivity to anti-angiogenic drugs and immunotherapy. The exploration of risk prediction model remains a big challenge to the selection of precision medicine for KIRC patients. New evidence suggests that RCC cells with high grade exhibit high levels of RIPK1 and RIPK3, which are more susceptible to necroptosis triggered by TNF receptor 1 26. Necroptotic pathway is involved in tumor necrosis 27, contributing to about half of the necrosis in head and neck squamous cell carcinoma 28. In KIRC, necrosis is a common pathological phenomenon correlating with invasive phenotypes and poor prognosis 29. In addition, necroptosis is correlated with microvascular invasion which has potential prognostic value in RCC 30. These evidence leads to the speculation that tumor spontaneous necroptosis is associated with adverse clinical outcome of KIRC. As a cancer type of high immunogenicity, KIRC microenvironment exhibits a high-level T cell infiltration 31. Within the spectrum of immunogenic cell death and drug resistance, necroptosis targeting has emerged as a potent anti-cancer therapeutic strategy 32, 33. Therefore, this study explored the potential impact of NRGs signature on KIRC microenvironment and its corresponding therapeutic response.

We firstly screened out the differentially expressed NRGs with prognostic values in KIRC, from which we constructed the novel risk signature consisting of three NRGs (FASLG, TLR3, and ZBP1). KIRC patients in high-risk group exhibited the upregulated expressions of FASLG and ZBP1 but a downregulated TLR3 expression. The pathway of Fas and Fas Ligand (FasL, FASL, FASLG) can initiate necroptosis upstream of RIPK3 and MLKL in renal tubular epithelial cells 34. The cell surface FasL induces Fas-mediated killing, while autocrine secretion of soluble FasL can protect RCC cells from cytotoxic lymphocytes killing 35. Previous studies indicated that FasL overexpression contributes to immune escape and associates with a poor prognosis in RCC 36, 37. As the toll-like receptor (TLR) family member, TLR3 has attracted most attention in its immune functional role, and prior studies report its high expression in immune and epithelial cells. In recent years, TLR3 overexpression is also found in multiple cancer types, but increasing evidence reveals that in tumorigenesis and progression, the dual or contradictory roles of TLR3 correlate with heterogeneous tumor cells and complex microenvironment 38. TLR3/TICAM-1 axis induces necroptosis via RIPK3 and MLKL 39. Damage-associated molecular patterns (DAMPs) released from necrotic cells, such as double-stranded RNA (dsRNA), can activate TLR3 and subsequently lead to proinflammatory response. TLR3 expression in human cancers is closely related to clinical characteristics, prognosis, metastasis, and therapy resistance 38, 40. A study revealed that TLR3 is frequently overexpressed in both primary and metastatic KIRC 41. ZBP1 interacts with RIPK3 to mediate tumor necroptosis 42. The role of ZBP1 remains ambiguous in tumor progression and metastasis. Recent research indicated that ZBP1 is highly increased in late stage of mouse and human tumors, and ZBP1 deletion inhibits tumor metastasis in pre-clinical cancer models 43. The expression of ZBP1 is increased in human cancers such as ovarian cancer and colon cancer, which is also associated with poor prognosis 44, 45. Interestingly, we also validated the significant upregulation of FASLG, TLR3, and ZBP1 expressions through TCGA, GEO datasets, and IHC staining samples from our center. The risk score calculated based on the three-NRGs signature could independently predict survival outcome in patients with KIRC.

The tumor immune microenvironment of KIRC has a high degree of immune cells infiltration with various immunomodulatory molecules 46, which may critically impact the immunotherapeutic resistance and efficacy 47. Based on the score comparisons of immune cell types and immune-related functions, we found that high-risk group with shorter OS possessed higher scores in macrophages, CD8 positive T cells, T cell co-inhibition and co-stimulation, suggesting an unbalanced and dynamic immune regulation in KIRC progression. Furthermore, the increased expressions of T cell exhaustion markers caused by persistent antigenic stimulation can lead to the functional loss of CD8 positive T cells 48. Consistently, our study indicated the elevated expressions of immune checkpoint genes, including CTLA-4, LAG-3, PD-1, and SIGLEC15. The enriched analysis also revealed the critical role of immunomodulatory functions in KIRC progression, such as abnormal proportion of CD8 positive T cells, T helper 1 type immune response. Consistent with previous studies 49, 50, our study found that cellular immune response was active in KIRC of the high-risk group. However, the overall effect of immunogenic cell death (ICD) may be limited by the defense mechanisms of tumor, such as PD-1 pathway. Interestingly, experimental evidence indicates the crosstalk between necroptosis and complement-dependent cytotoxicity acting on cancer cells 51. Therefore, the combination of targeting necroptosis-related ICD and immunotherapy could be a novel therapeutic direction for KIRC.

Based on the TCGA dataset analyses, a novel cluster named mixed subgroup was identified and exhibited striking overexpression of mitochondrial DNA (mtDNA) 52. Mixed subgroup affiliation was associated with a highly significant shorter OS in KIRC 52. mtDNA has been reported to release to the cytosol of cancer cells that bear necroptosis and ZBP1 senses the cytosolic mtDNA for the initiation of cancer necroptosis under glucose deprivation or stress condition 53-55. Furthermore, endogenous oxidatively damaged mtDNA can induce proinflammation in epithelial cells through binding to ZBP1 56. In our model, ZBP1 has the highest hazard ratio among three NRGs. Based on the evidence of necroptosis and ZBP1 expression, we deduce a close affiliation between the mixed subgroup and the high-risk group in our NRGs risk model. In addition, we analyzed the expression of mitochondrial and angiogenesis-related genes in our NRGs risk model. The high-risk group displayed significantly higher levels of mitochondrial gene expressions and lower levels of angiogenesis gene expressions compared to the low-risk group (**Figure S3, S4**). This evidence strengthens the role of the high-risk group in our model, for the upregulation of mitochondrial genes is associated with tumor growth and cancer multidrug resistance 57. On the other hand, lower angiogenesis gene expression is associated with a poor prognosis in advanced RCC 58.

To promote the clinical transformation of the NRGs signature, we further identified the sensitive FDA-approved drugs for multiple cancer cell types with higher expressions of FASLG, TLR3, and ZBP1. Increased expression of FASLG is associated with sensitivity of cancer cells to oxaliplatin, ribociclib (LEE-011) and palbociclib. Oxaliplatin plus gemcitabine is a combination-therapeutic strategy for advanced KIRC 59, while the combination of oxaliplatin and decitabine is a promising option to treat KIRC 60. Ribociclib and palbociclib are orally administered small-molecule inhibitors of CDK4/6, which function in synergy with other drugs to suppress KIRC in preclinical models 61-63. The upregulated expression of TLR3 was associated with sensitivity of cancer cells to erdafitinib (JNJ-42756493) and duvelisib (IPI-45), but it was associated with increased drug resistance to tyrothricin. Erdafitinib shows preliminary clinical activity in advanced solid tumors with genomic alterations of Fibroblast Growth Factor Receptor (FGFR) pathway, which is also a crucial target for KIRC treatment 64, 65. Moreover, tyrothricin complex contains gramicidin A as a potential agent for KIRC therapy 66, 67. PI3K/AKT and VHL/HIF pathways are closely connected to form a large signaling network contributing to KIRC 68. In clinical practice, a PI3K inhibitor, not duvelisib, shows anti-cancer effects on RCC 69. The upregulation of ZBP1 expression was correlated with sensitivity of cancer cells to ceritinib (LDK-378), alectinib, and brigatinib. Recently, clinical case studies demonstrated that these small molecule inhibitors of anaplastic lymphoma kinase are promising agents for RCC precise treatment 70, 71.

Finally, a NRGs nomogram was constructed to predict 1/3/5/7/10-year OS by combining NRGs risk score and clinicopathological features, showing a favorable agreement between the actual and predicted probability. However, this study still exists some limitations. Firstly, our study is retrospective in nature, requiring the use of prospective studies to validate the findings. Secondly, larger KIRC cohorts are still required to test the applicability of the necroptosis related signature. Thirdly, the novel molecular mechanisms of NRGs in KIRC need to be investigated via *in vivo* and *in vitro* experiments.

## Conclusions

Based on the systematic analyses, we identified and verified a three-NRGs (TLR3, FASLG, ZBP1) risk signature with good performance in predicting survival outcome, immune microenvironment, and therapeutic sensitivity in KIRC. These findings offer molecular-level evidence to the critical role of necroptotic process in regulating immune microenvironment and therapeutic response in KIRC.

## Supplementary Material

Supplementary figures.

## Figures and Tables

**Table 1:** Characteristics of train cohort, test cohort, and entire cohort

	Train cohort, N = 257	Test cohort, N = 256	Entire cohort, N = 513	P*
	Number (%)	Number (%)	Number (%)	
**Age**				0.319
≤65	165 (64.2)	175 (68.4)	340 (66.3)	
>65	92 (35.8)	81 (31.6)	173 (33.7)	
**Gender**				0.476
Female	92 (35.8)	84 (32.8)	176 (34.3)	
Male	165 (64.2)	172 (67.2)	337 (65.7)	
**Grade**				0.778
1-2	115 (44.7)	116 (45.3)	231 (45.0)	
3-4	137 (53.3)	137 (53.5)	274 (53.4)	
Unknown	5 (2.0)	3 (1.2)	8 (1.6)	
**Stage**				0.312
I/II	160 (62.3)	152 (59.4)	312 (60.8)	
III/IV	97 (37.7)	102 (39.8)	199 (38.8)	
Unknown	0 (0.0)	2 (0.8)	2 (0.4)	
**Survival**				0.935
Live	172 (66.9)	172 (67.2)	344 (67.1)	
Dead	85 (33.1)	84 (32.8)	169 (32.9)	

*Statistical analysis in age, gender, grade, stage, and survival between train cohort and test cohort.
