# Supplementary material for: A Novel Prognostic Predictor of Immune Microenvironment and Therapeutic Response in Kidney Renal Clear Cell Carcinoma based on Necroptosis-related Gene Signature
Source: Int J Med Sci. 2022 Jan 24;19(2):377–92. doi: 10.7150/ijms.69060 (PMC8795799; doi:10.7150/ijms.69060)
Supplement: Supplementary file 1 — Supplementary figures. [file ijmsv19p0377s1.pdf]

Figure S1

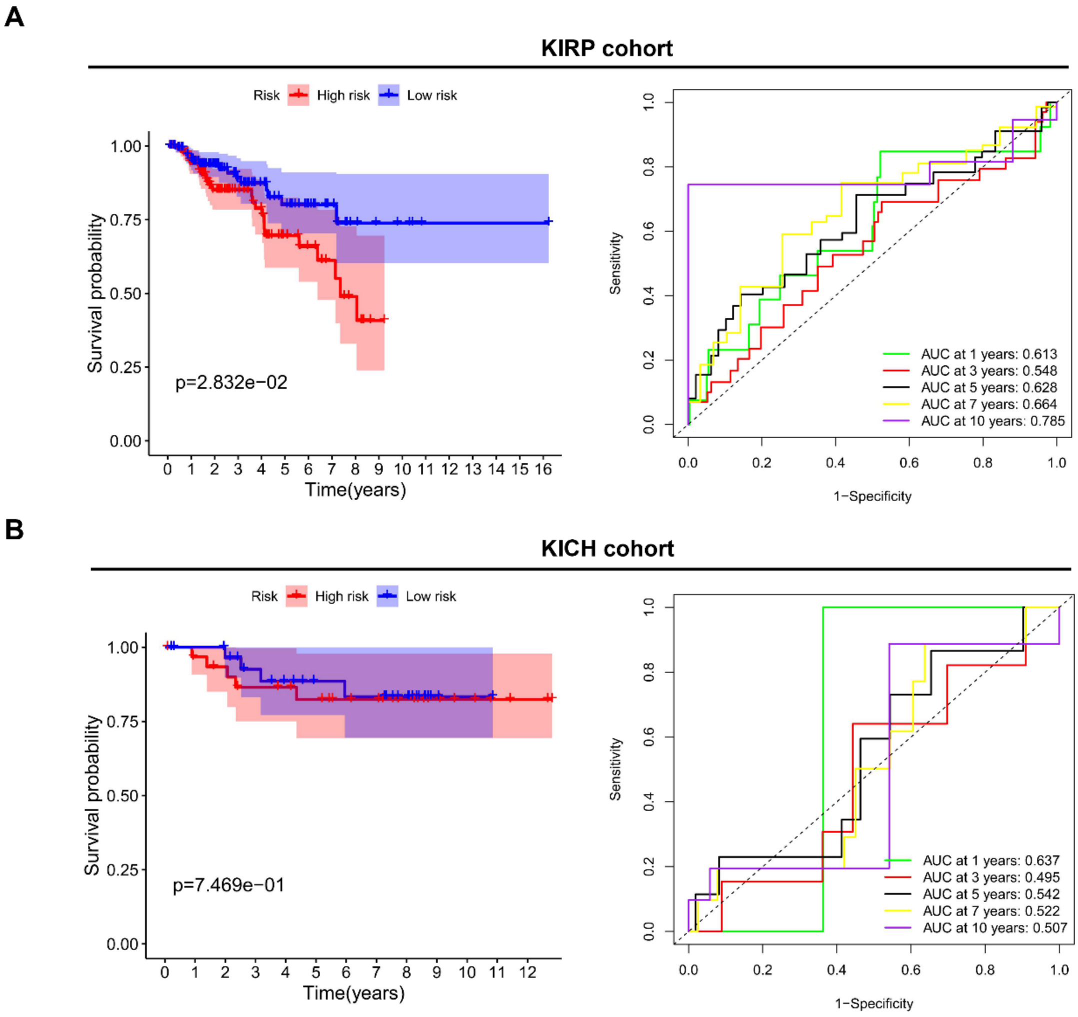

Figure S1 The prediction of KIRP and KICH cohorts by NRGs risk model.
(A) The K-M curve and time-dependent ROC curve for OS in KIRP cohort. **(B)** The K-M curve and time-dependent ROC curve for OS in KICH cohort. Abbreviations: KIRP, kidney renal papillary cell carcinoma; KICH, kidney chromophobe; NRGs, necroptosis-related genes; K-M, Kaplan-Meier; OS, overall survival.

Figure S2

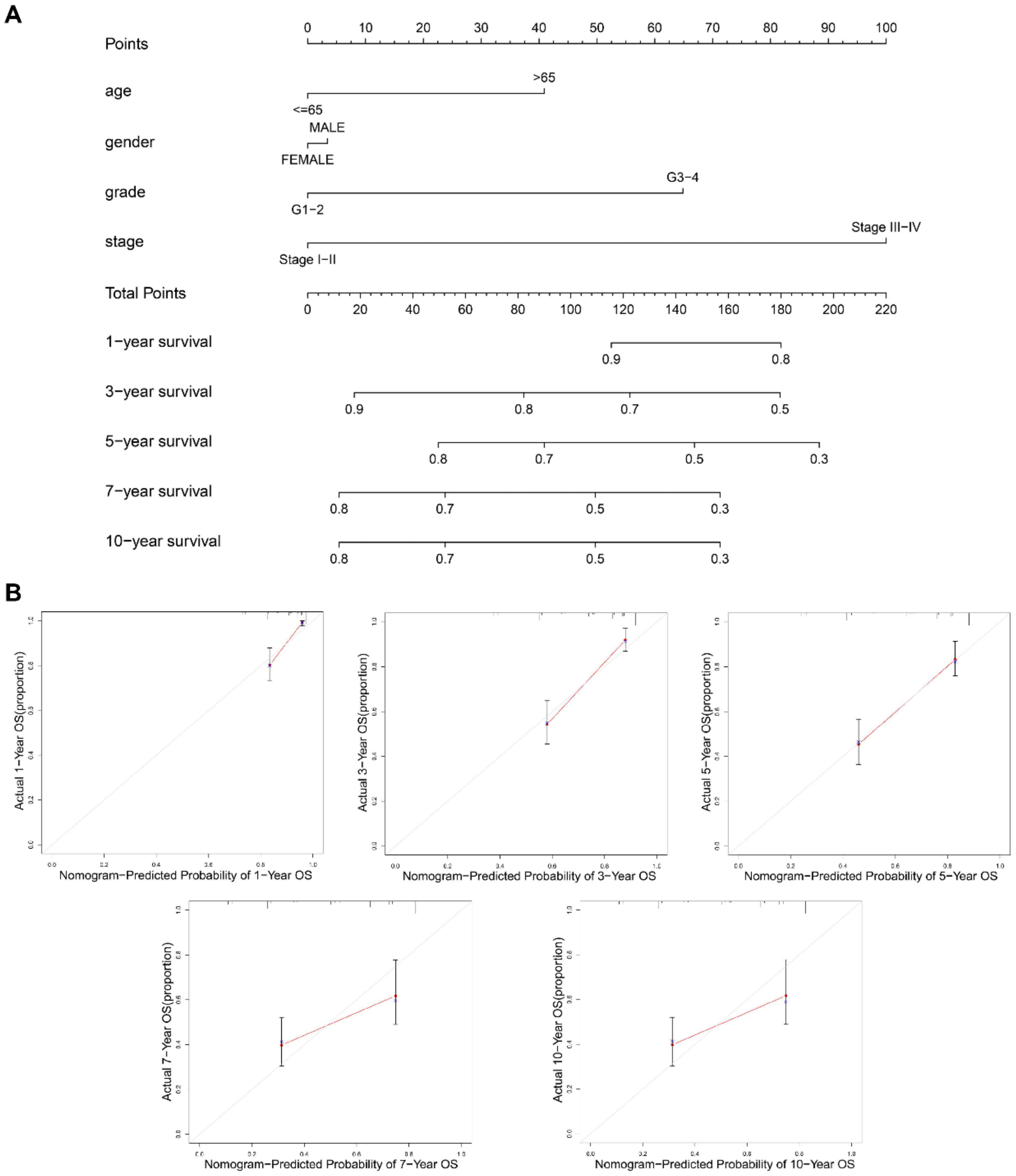

Figure S2. The construction of a nomogram for predicting survival.

(A) A nomogram including only clinicopathological features was constructed to predict 1/3/5/7/10-year OS. **(B)** The calibration plots for predicting 1/3/5/7/10-year OS based on the NRGs nomogram.

Figure S3

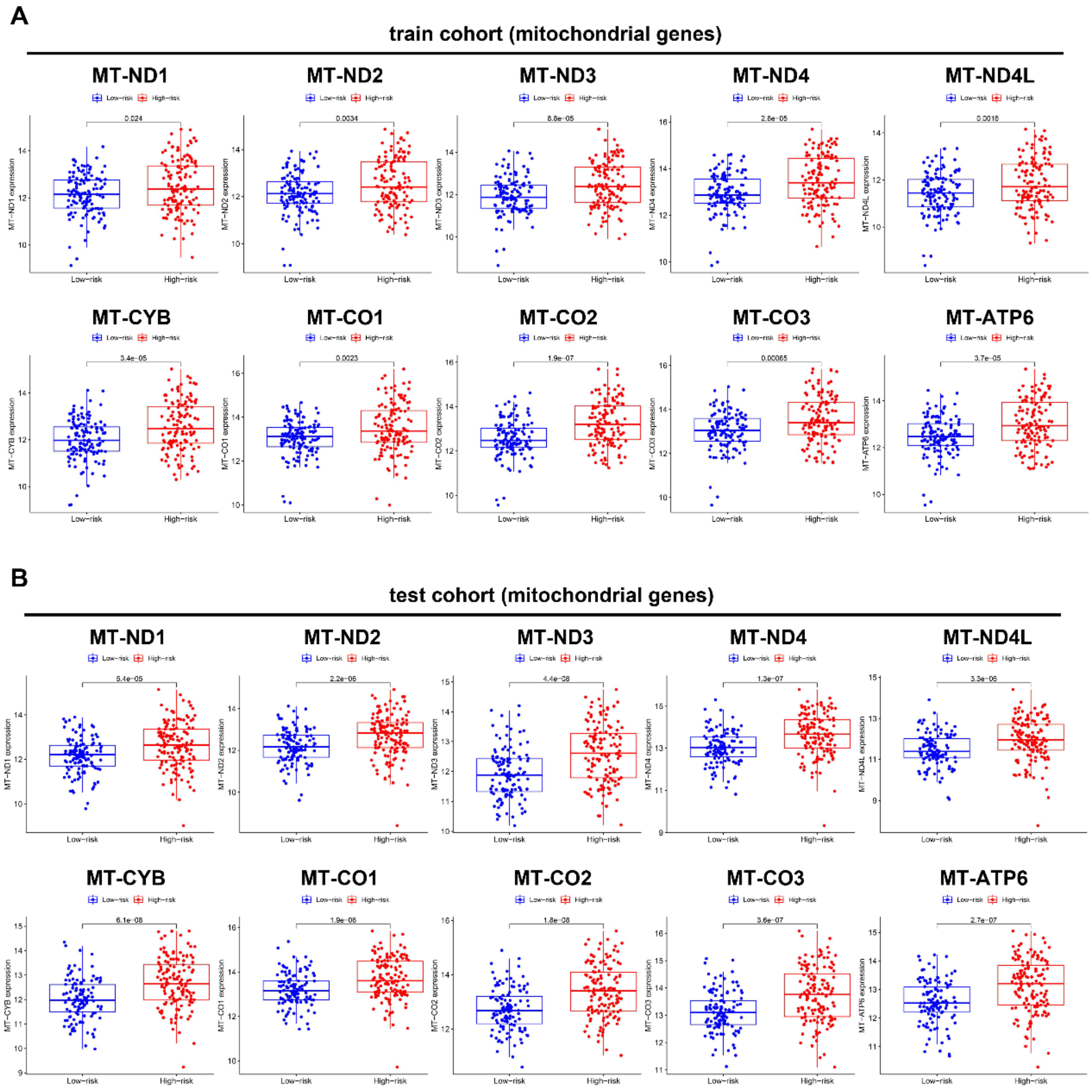

Figure S3. The comparison of mitochondrial gene expressions.

The expressions of mitochondrial genes, including MT-ND1, MT-ND2, MT-ND3, MT-ND4, MT-ND4L, MT-CYB, MT-CO1, MT-CO2, MT-CO3, and MT-ATP6, were significantly higher in the high-risk group both in **(A)** train cohort and **(B)** test cohort.

Figure S4

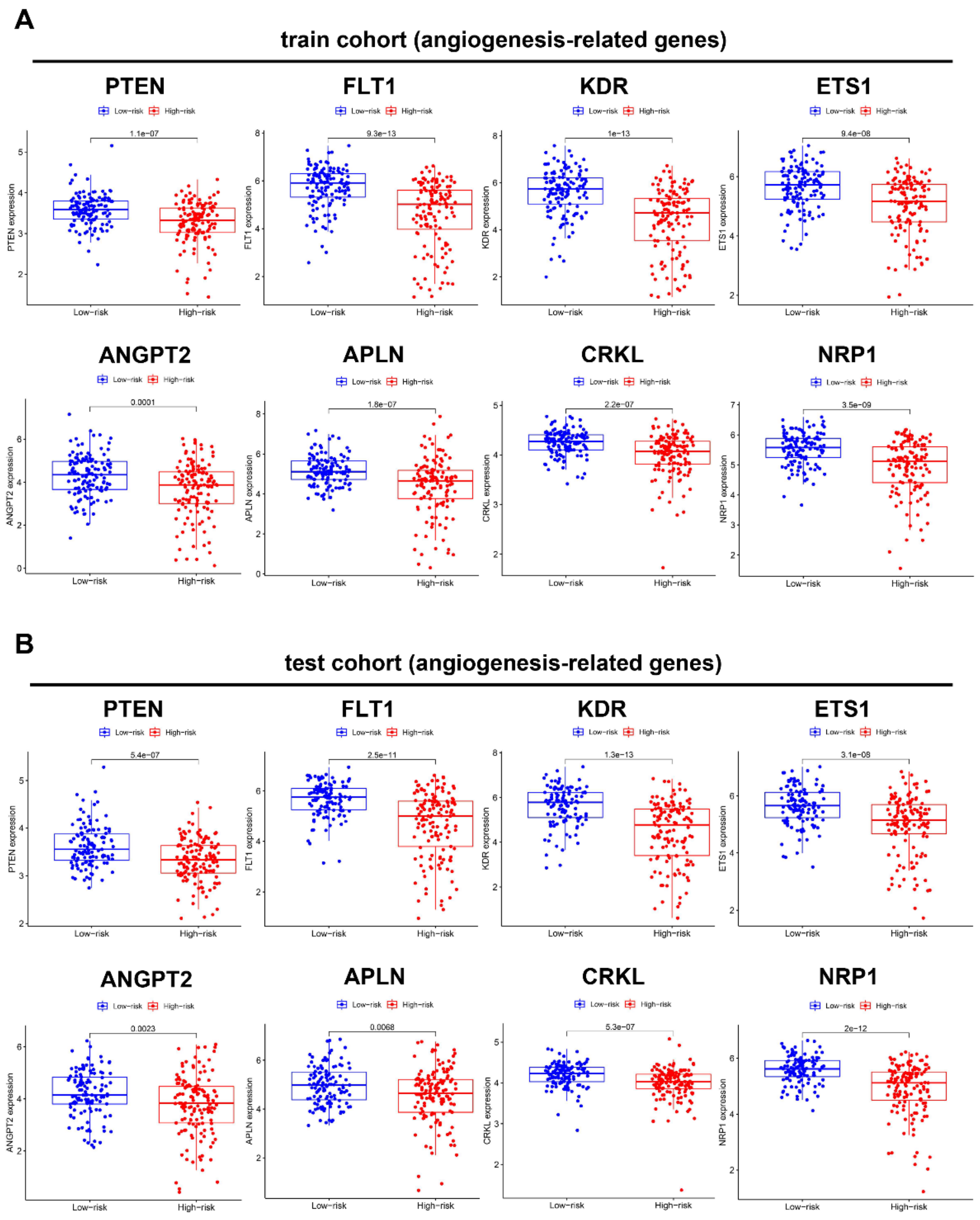

Figure S4. The comparison of angiogenesis-related gene expressions.
The expressions of angiogenesis-related genes, including PTEN, FLT1, KDR, ETS1, ANGPT2, APLN, CRKL, and NRP1, were significantly lower in the high-risk group both in **(A)** train cohort and **(B)** test cohort.
